# Repetitive transcranial magnetic stimulation (rTMS) for comorbid major depressive disorder and hoarding disorder: An open label pilot study

**DOI:** 10.1016/j.jad.2026.121814

**Published:** 2026-04-16

**Authors:** Jessica J. Zakrzewski, Lawrence G. Appelbaum, Cory R. Weissman, Yinming Sun, Catherine R. Ayers, Zafiris J. Daskalakis, Elizabeth W. Twamley

**Affiliations:** a Department of Psychiatry, UC San Diego, La Jolla, CA, United States; b Center of Excellence for Stress and Mental Health, VA San Diego Healthcare System, San Diego, CA, United States; c Mental Health Care Line, VA San Diego Healthcare System, San Diego, CA, United States

**Keywords:** rTMS, Depression, Hoarding, Treatment, Stimulation

## Abstract

**Background:**

Hoarding Disorder (HD) is characterized by persistent difficulty discarding possessions due to a strong urge to save them, resulting in excessive clutter that compromises functioning. Despite the common prevalence and profound consequences of HD, there are no FDA approved treatments. Individuals with HD often have comorbid major depressive disorder (MDD). This study examined the feasibility and initial efficacy of repetitive transcranial magnetic stimulation (rTMS) for HD patients with comorbid MDD.

**Methods:**

Participants with moderate or severe MDD and co-morbid HD underwent daily neuronavigated intermittent theta burst stimulation (iTBS) rTMS to the left dorsolateral prefrontal cortex for 20 sessions over four weeks. Assessments of symptoms and neuropsychological performance were collected at pre-treatment, post-treatment, and 1-month follow up.

**Results:**

Eleven individuals enrolled in the study (6 women; mean age 52). One participant dropped out after six sessions. Following iTBS treatment, depression symptoms on the Hamilton Depression Rating Scale-17 were reduced by 43% at post-treatment and 48% at follow-up. Hoarding symptoms on the Hoarding Rating Scale were reduced by 28% at post-treatment and 29% at follow-up, with the greatest reductions in difficulty discarding. Neuropsychological performance improvements were seen in attention/working memory, visual scanning, processing speed, and verbal learning (*p* < 0.05).

**Conclusions:**

This is the first clinical trial of rTMS for MDD + HD and demonstrates preliminary feasibility and clinical improvement and the potential role for rTMS in HD treatment. Using rTMS to treat MDD in individuals with HD may open new avenues of care for this severe and undertreated disorder.

## 1. Introduction

Hoarding Disorder (HD) is defined by the significant and persistent difficulty in discarding objects, regardless of their value, with clutter, distress, and psychosocial impairment as consequences (American Psychiatric et al., 2013). Although the most recent pooled lifetime prevalence of HD is estimated at 2.5%, both the prevalence and the severity of symptoms increase with age to a prevalence of nearly 6% in individuals 55 years and older (Cath et al., 2017; Postlethwaite et al., 2019). There are significantly elevated rates of psychiatric comorbidities in HD, with 50–80% of individuals with HD having co-occurring major depressive disorder (MDD) and 20–30% having obsessive-compulsive disorder (OCD), post-traumatic stress disorder (PTSD), generalized anxiety disorder, and/or attention-deficit hyperactivity disorder (Archer et al., 2019; Frost et al., 2011). In addition, cognitive impairments have been identified in HD samples, particularly on visually-based tasks and tests of executive functioning (Kassel et al., 2024; Mackin et al., 2016; Zakrzewski et al., 2020).

There are currently no FDA-approved treatments for HD. The gold standard treatment for HD is cognitive behavioral therapy (CBT) with exposure therapy for discarding possessions (Rodgers et al., 2021). Currently, only one-third of individuals respond to treatment and post-treatment severity levels are often still above clinical cut-offs for HD (David et al., 2022). For individuals who do respond to treatment, defined by reductions in the Saving Inventory-Revised (SI-R) (Rodgers et al., 2021), response rates vary between 18 and 38% across studies, with a 25% reduction in symptoms on average (David et al., 2022; Rodgers et al., 2021). Drop-out rates for HD treatment trials are also high, with lack of insight, poor emotion regulation, and avoidance as possible reasons for attrition as well as evidence that comorbidities such as MDD reduce the effectiveness of treatment (Ayers et al., 2014; David et al., 2022; Rodgers et al., 2021; Tolin et al., 2015). Current pharmacological treatment studies are limited to case series or open label studies of antidepressant or stimulant medications, with no randomized controlled trials of any medication for HD (Thompson et al., 2017). Furthermore, low provider confidence in identifying and treating HD (Stanley et al., 2017; Zakrzewski et al., 2024) in addition to the lack of reliable effects from behavioral treatments, and any current FDA-approved treatments, there is a significant need for new treatment approaches (David et al., 2022; Stanley et al., 2017).

Repetitive transcranial magnetic stimulation (rTMS) is an effective, FDA-approved treatment for MDD. rTMS is a noninvasive procedure in which an electromagnetic coil is placed against the scalp and electromagnetic pulses are delivered to stimulate a specific brain area. Meta-analytic findings indicate robust anti-depressant effects of rTMS (Sehatzadeh et al., 2019). rTMS for MDD targets the left dorsolateral prefrontal cortex (dlPFC) and is thought to modulate neural activity in this region and other highly connected brain areas, thereby improving mood regulation and reducing MDD symptomatology (Chen et al., 2020; Sonmez et al., 2019). Specifically, alterations in functional connectivity between the dlPFC and the subgenual cingulate (SGC) have been associated with improved treatment response, leading to the development of individualized targeting approaches based on these connectivity patterns (Cash et al., 2019). In addition to improvements in targeting, a new stimulation pattern, intermittent theta burst stimulation (iTBS), delivers patterned bursts of magnetic pulses in a much shorter time while producing comparable or even superior antidepressant effects (Blumberger et al., 2018). Beyond mood symptoms, the dlPFC plays a central role in executive functioning and has been associated with improvements in cognitive functioning in individuals with depression (Brunoni and Vanderhasselt, 2014; Fu et al., 2025; Hart et al., 2013). Additionally, rTMS for MDD has demonstrated efficacy in individuals with MDD and comorbid conditions including anxiety, PTSD, and chronic pain (Corlier et al., 2021; Liu et al., 2024).

MDD and HD share symptoms of amotivation, concentration difficulties, difficulty with emotion regulation, indecision, and fatigue (Levy et al., 2019; Raines et al., 2016; Tolin, 2023) (Archer et al., 2019). Given these areas of overlap, stimulation to the left dlPFC for individuals with comorbid MDD + HD may have effects on difficulty discarding, emotion dysregulation, indecision, and/or motivation. Furthermore, it may also improve executive functioning, a key area of cognitive dysfunction for many with HD and thought to be associated with difficulty discarding possessions (Ayers and Dozier, 2015; Tolin, 2023) However, there have been no clinical trials using rTMS treatment for individuals with MDD + HD. As such, we conducted the first open label pilot clinical trial of rTMS treatment for MDD + HD (NCT05985356). We used the latest advancements of individualized targeting for depression and iTBS treatment to maximize potential treatment response (Cash et al., 2019; Fox et al., 2012). We hypothesized that rTMS would be feasible and result in improved depression symptoms in MDD + HD, as well as yielding improvements in HD symptoms and cognitive functioning.

## 2. Methods

### 2.1. Overview

Participants were initially recruited from a waitlist for HD treatment in the San Diego, California area. However, participants also found the study online via clinicaltrials.gov and through word of mouth in a local clutter anonymous group. Screenings were performed and if all criteria were met, consent and baseline assessment were scheduled. After providing written informed consent, participants completed baseline assessments, including clinical interviews, self-report measures, a short neuropsychological assessment, and MRI scans. Treatment was scheduled each weekday for a total of 20 sessions over four consecutive weeks. Self-reported HD and MDD symptoms were collected weekly. All self-report measures and neuropsychological tests were repeated following treatment. A final assessment that consisted of clinical interviews, self-report measures, and neuropsychological assessment was completed 4-weeks following treatment.

### 2.2. Inclusion/exclusion criteria

Participants were required to be between ages 18–70, meet criteria for HD and MDD, have significant levels of MDD (Hamilton Depression Rating Scale-17 (HAMD) ≥18 and Patient Health Questionnaire-9 (PHQ-9) ≥10) and HD (SI-*R* ≥ 42 and Hoarding Rating Scale (HRS) ≥14) symptoms, and be stable on psychiatric medications for 6 weeks. Participants were excluded if they had a history of neurological conditions, significant brain injury, were currently pregnant, had active substance use, psychosis, mania, suicidal ideation, or homicidal ideation, were on anticonvulsant medication, were on a benzodiazepine equivalent to 2 mg lorazepam or greater, or had previously failed rTMS, iTBS, or electroconvulsive therapy. Participants were also screened for both MRI and rTMS safety.

### 2.3. Clinical assessments

Diagnostic interviews included validation of HD diagnosis with the Structured Interview for Hoarding Disorder (SIHD) (Nordsletten et al., 2013), a structured clinical interview reviewing all key DSM-5 diagnostic criteria. Comorbid psychiatric diagnoses were assessed using the Diagnostic Interview for Anxiety, Mood, and OCD and Related Neuropsychiatric Disorders (DIAMOND) (Tolin et al., 2016), a semi-structured clinical interview designed to assess for common mood, trauma, and OCD-related disorders (Tolin et al., 2016). Current depression symptoms were assessed through clinician interview (HAMD) and PHQ-9. Current HD symptomatology was assessed with the SI-R and HRS. Depression and HD symptoms were assessed weekly throughout treatment via the PHQ-9 and HRS.

### 2.4. Neuropsychological assessment

Neuropsychological tests were selected to focus on frequent areas of impairment seen in both MDD and HD including executive functioning, processing speed, and verbal learning and memory. Tests included the Wide Range Achievement Test-4 (WRAT-4) Reading (Wilkinson and Robertson, 2006) to estimate intellectual ability; Delis-Kaplan Executive Functioning System (D-KEFS) Trail-Making Test (TMT) (Delis et al., 2001) to measure visual, psychomotor, and executive processes; D-KEFS Color-Word Interference (Delis et al., 2001) to measure inhibition and switching; Weschler Adult Intelligence Scale-IV (WAIS-IV) Digit Span (Wechsler, 1955) as a measure of verbal working memory; WAIS-IV Processing Speed Index (Coding and Symbol Search) (Wechsler, 1999) as a measure of visual scanning and information processing speed; and the Hopkins Verbal Learning Test-Revised (HVLT-R) to assess verbal learning and memory (Shapiro et al., 1999). Alternate forms were used when available and primary metrics for each measure were converted to standardized scores using published normative data.

### 2.5. Neuroimaging and target generation

A 3-T Siemens Prisma MRI scanner with a 32-channel phased-array head coil (Siemens, Erlangen, Germany) was used to obtain T1-weighted MPRAGE (TR 2400 ms; TI 1000 ms; TE 2.14 ms; FOV 256 mm; flip angle 8°; 208 slices; voxel size = 1 mm isotropic) and T2-weighted SPACE (TR 3200 ms; TE 564 ms; FOV 256 mm; 208 slices; voxel size 1 mm isotropic) scans, as well as eyes-open resting state functional MRI (fMRI) scans at the Center for Functional MRI in the University of California, San Diego. The resting state fMRI scans were obtained with an echo-planar multi-band, multi-echo sequence (TR 1330ms; TE1 12.6ms, TE2 29.5ms, TE3 46.4 ms, and TE4 63.3ms; FOV 216 mm; flip angle 67°; AP phase encoding direction; in-plane acceleration factor 2; multi-band acceleration factor 4; 60 slices; 2.5mm isotropic voxels) lasting for 313 volumes or 6 min 56 s. Three resting state scans were collected and Fmriprep (version 23.0.2) was used for preprocessing the structural and fMRI scans (Esteban et al., 2019). Individual echo fMRI images were optimally combined using T2* estimates. The resulting combined multi-echo image was subsequently processed with ICA-AROMA for artifact rejection and subject to global signal regression (DuPre et al., 2021; Pruim et al., 2015). Each resting state scan was processed separately before being co-registered and concatenated. The target location was determined as the location within the left dlPFC with the highest anti-correlation with subgenual cingulate cortex (SGC) based on established techniques reported in the literature (Fox et al., 2012).

### 2.6. Electric-field modeling and resting motor threshold

Once the optimal target voxel was determined in the MRI volumetric space, the coordinates were used to generate a TMS coil position on the scalp that produces the largest electric field (*E*-field) around the center coordinate. E-field modeling accounted for changes in electrical conductivity between cerebrospinal fluid, gray matter, and white matter, as well as variations in the subject’s dlPFC gyral shape and orientation, which influence current distribution depending on coil positioning. The E-field optimization was done with the targeting and analysis pipeline (TAP) software (Dannhauer et al., 2022). TAP searched through a range of possible TMS coil placements including pitch, roll, and yaw, with minimal shifts in the target center coordinate by comparing their corresponding simulated E-field generated with SimNIBS (Saturnino et al., 2019). Individual target coordinates are presented in Supplementary Table 1. The stimulation intensity in terms of the maximum stimulator output (MSO%) was set to achieve 114 V/m at the target center with the specific stimulator and coil used (i.e., MagVenture X100 stimulator with a A/P Cool-B65 coil), which was based the average E-field generated at the motor cortex for 120% of the resting motor threshold (RMT) for a reference population (Beynel et al., 2020; Dannhauer et al., 2022). For safety, RMT was also determined using stochastic approximation MT (Wang et al., 2023) to confirm no E-field estimates were above 120% calculated RMT.

### 2.7. TMS treatment procedures

Treatment was delivered once per weekday for a total of 20 sessions over the course of four-to-five weeks, dependent on holidays. The Brainsight neuronavigation system (Rogue Research, Montreal, Quebec) was used for targeting for all participants. Individual target coordinates, orientation vectors for the left dlPFC, and the individual’s reconstructed scalp and cortical surfaces from their T1 image were loaded into the system. Physical head and scalp coordinates were verified at the start of each session and confirmed to be within 3 mm of target alignment prior to treatment and throughout as necessary to maintain target integrity. iTBS was delivered as 3-pulse 50-Hz bursts, applied at 5 Hz (i.e., 50 Hz burst of 3 pulses delivered every 200 ms) with a 2-s train of TBS repeated every 10 s (i.e., 2 s of TBS followed by an 8-s rest). The total number of pulses was 600 per session (Beynel et al., 2020; Dannhauer et al., 2022).

### 2.8. Data analysis

Statistical analysis was performed using SPSS version 29. Visual inspection, rates of change, and one-way repeated measures analysis of variance (rmANOVA) were used to assess changes in clinical symptoms and neuropsychological test performance across time (baseline, post-treatment, and 1-month follow up). Greenhouse-Geisser correction was used when the assumption of sphericity was violated. Clinically significant change for MDD symptoms was defined as a 50% reduction from baseline on the HAM-D or PHQ9. Clinically significant change for HD symptoms was defined as a 20-point or greater reduction on the SIR, with those falling below the clinical cutoff of 42 defined as recovered (Norberg et al., 2021). While there is no marker for clinically significant change for the HRS, the clinical cut-off for HD is 14 points or more (Tolin et al., 2010). Pearson correlations were conducted to determine associations between MDD and HD symptom change.

## 3. Results

### 3.1. Feasibility/acceptability

**Fig. 1.**
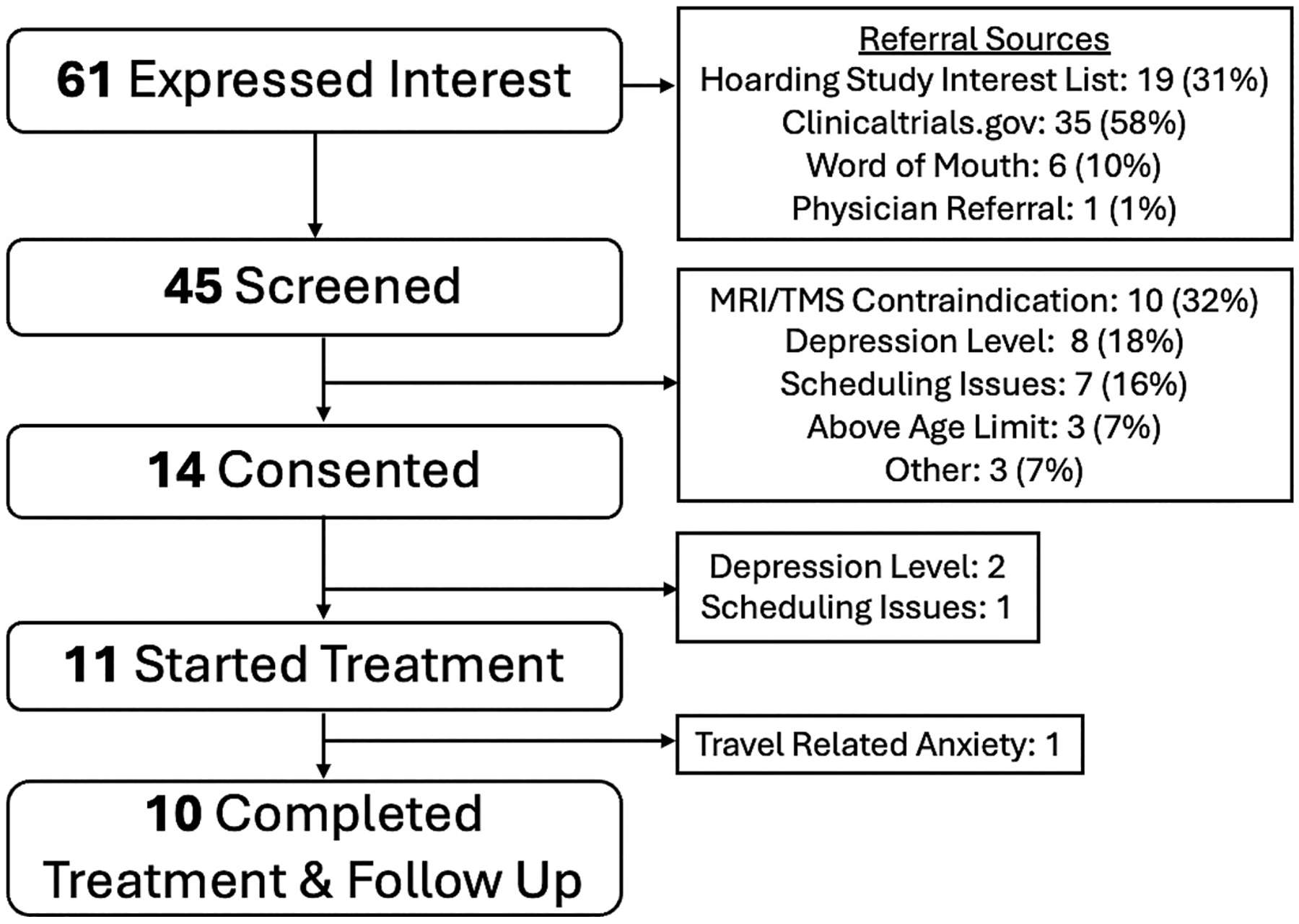
CONSORT Diagram.

Fourteen individuals consented to participate (Fig. 1). One participant did not complete a baseline assessment due to scheduling issues, and two were excluded post-consent due to low levels of current depression symptoms during clinical interviews. The remaining 11 participants initiated treatment. One participant dropped out after six sessions due to transportation-related anxiety due to comorbid PTSD. The remaining 10 out of 11 participants (91%) completed treatment and all assessment measures. Half of the sample missed no scheduled treatment days, while the other half had to reschedule an average of 2 days (sd = 3.1; range = 9) with sickness, traffic, and family emergencies reported as reasons for rescheduling. No significant unexpected adverse events occurred. Anticipated side effects reported included headache lasting no longer than a day (7/10), jaw/face pain during treatment (5/10), and anxiety about treatment (1/10).

### 3.2. Participant characteristics

Of the eleven individuals who initiated treatment, 73% identified as White (8 White; 1 Asian/Pacific Islander; 2 Hispanic/Latino/a) with a nearly even split of sex (5 men/6 women). There was a wide range in age, with a mean of 52 years (range 27 to 68), and years of education (mean = 14, range 6–18). The majority of participants were taking some form of psychiatric medication (6/11, 55%) including 4/11 on an antidepressant, 3/11 on a stimulant, 1/11 on an antipsychotic, and 2/11 on a benzodiazepine, with all medication doses confirmed stable for six weeks prior and throughout treatment. Some form of psychotherapy was concurrent for 4/11 (36%) of participants, although none were in HD-specific treatments. At baseline, all participants had at least moderate levels of depression symptoms and met clinical cutoffs for HD (Table 1). All participants had at least one comorbidity in addition to meeting criteria for MDD and HD; 5/11 meet criteria for generalized anxiety disorder, 5/11 for PTSD, 3/11 for attention deficit hyperactivity disorder, and 1/11 for OCD.

### 3.3. Clinical outcomes

**Fig. 2.**
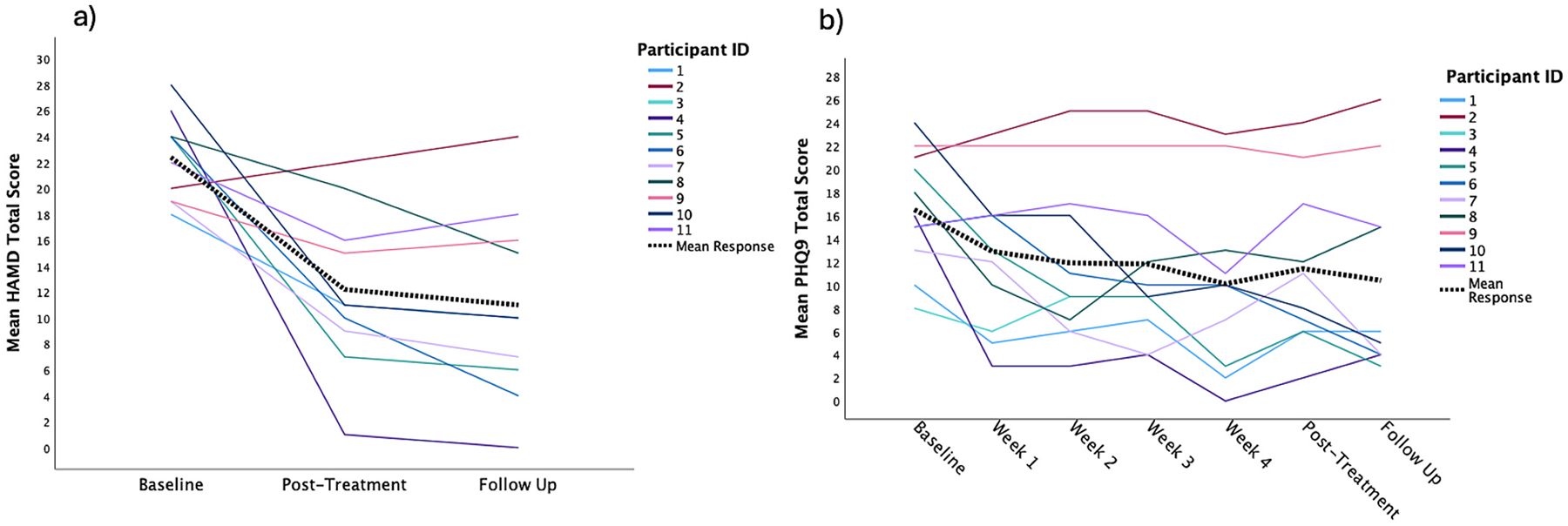
ID represents the same participant across figure. Figure on the left a) individual HAMD response across treatment timepoints including representation of mean response overall. Figure on the right b) individual PHQ9 response across treatment timepoints.

Following a 20-session course of daily, individualized iTBS, there were significant reductions in depression symptoms on both clinician and self-report symptom measures. On the HAMD there was significant main effect of time with a large effect size (Table 1). The mean reduction in symptoms from pre-to-post treatment was 43% (*mΔ* = 10.2 points, *sd* = 8.0, *p* = 0.003, *95% CI* = 2.8–17.6) and from pre-treatment to 1-month follow-up was 48% (*mΔ* = 11.4 points, *sd* = 9.2, *p* = 0.003, *95% CI* = 2.9–19.9) (Table 1; Fig. 2a). Five of the ten participants who completed treatment had a 50% reduction in depression scores at follow up and seven out of 10 had at least a 30% reduction in symptoms. On the PHQ-9 there was a significant main effect for time with a large effect size (Table 1). The mean symptom reduction from pre-to-post treatment was 34% (*mΔ* = 6.0 points, *sd* = 6.8, *p* = 0.022, *95% CI* = 1.1–10.9) and from pre-treatment to 1-month follow-up was 41% (*mΔ* = 7.0 points, *sd* = 7.9, *p* = 0.020, *95% CI* = 1.4–12.6) (Fig. 2b). On this self-report measure 4/10 had a 50% reduction at follow-up and 6/10 had at least a 30% reduction by follow up.

**Fig. 3.**
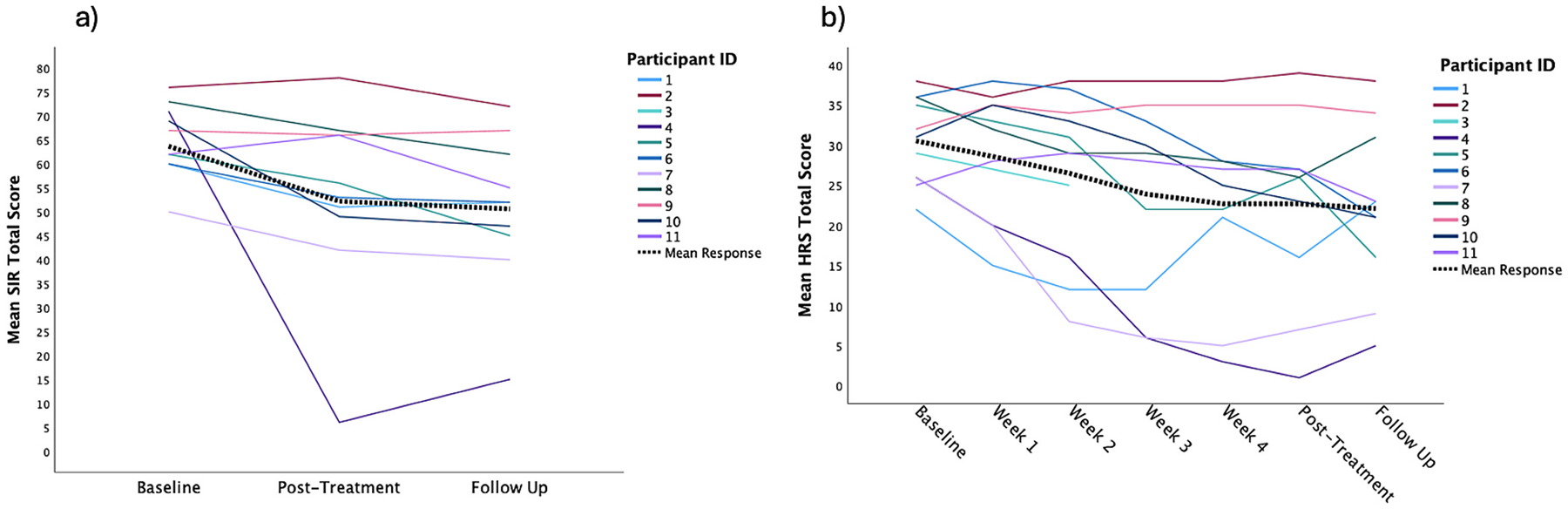
ID represents the same participant across figures. Figure on the left a) individual SIR response across treatment timepoints including representation of mean response overall. Figure on the right b) individual HRS response across treatment timepoints.

HD symptoms were reduced following rTMS. On the SIR, there was a significant main effect for time with a large effect size (Table 1). The mean symptom reduction from pre-to-post treatment was 19% (*mΔ* = 12.0 points, *sd* = 16.3, *p* = 0.045, *95% CI* = 0.3–23.7) and from pre-treatment to 1-month follow-up was 21% (*mΔ* = 13.6 points, *sd* = 13.9, *p* = 0.013, *95% CI* = 3.7–23.5) (Fig. 3a). Two individuals met criteria for recovered (SI-*R <* 42) following treatment (through 1-month follow up) as well as both meeting criteria for clinically significant change (≤ 20 points). In addition, 5/10 had a reduction of at least 15% in HD symptoms. Across all symptom cluster subscales of the SIR, there were significant main effects for time, with large effect sizes for two of the three subscales. Difficulty discarding scores reduced 20% at post-treatment (*mΔ* = 4.0 points, *sd* = 3.1, *p* = 0.003, *95% CI* = *1.8*–*6.2*) and 21% at follow up (*mΔ* = 4.2 points, *sd* = 2.9, *p* = 0.001, *95% CI* = 2.2–6.2); clutter scores reduced 21% at post-treatment (*mΔ* = 5.9 points, *sd* = 8.1, *p* = 0.046, *95% CI* = 0.1–11.7) and 26% at follow-up (*mΔ* = 7.1 points, *sd* = 6.8, *p* = 0.009, *95% CI* = 2.2–12.0). Although there were reductions in excessive acquisition scores of 14% at post-treatment (*mΔ* = 2.8 points, *sd* = 6.1) and 16% at follow-up (*mΔ* = 3.1 points, *sd* = 5.1), these were not statistically significant. On the HRS there was a significant main effect for time with a large effect size (Table 1). The mean symptom reduction was 28% (*mΔ* = 8.0 points, *sd* = 9.9, *p* = 0.020, *95% CI* = 1.6–14.4) from pre-to-post treatment and 29% (*mΔ* = 8.6 points, *sd* = 8.9, *p* = 0.014, *95% CI* = 2.2–15.0) from pre-treatment to 1-month follow-up (Fig. 3b). On this self-report measure we saw nearly all of the participants (7/10) had at least 25% reduction in scores at post-treatment and 2/10 had scores below the clinical cutoff of 14 points for HD at post-treatment and follow-up. The two individuals who had clinically significant change in HD symptoms also had a 50% reduction in depression symptoms. Overall, change in HD and MDD symptoms using the SIR and HAMD significantly correlated at post-treatment (*r* = 0.82, *p* = 0.004) and follow-up (*r* = 0.77, *p* = 0.009). The shorter scales of HRS and PHQ were modestly correlated at post-treatment (*r* = 0.58, *p* = 0.077) and more strongly correlated at follow-up (*r* = 0.78, *p* = 0.007).

### 3.4. Neuropsychological outcomes

Overall estimated IQ based on WRAT-4 Reading performance was in the normal range, as were all standardized mean baseline scores across all tests (Supplementary Table 2). In examining each test by time, we found significant effects for time on performance on visual scanning, processing speed, inhibition, attention/working memory, and verbal learning (Table 2). In examining patterns of significant performance improvement, visual scanning performance significantly improved both at post-test (*p* = 0.031) and 1-month follow-up (*p* = 0.003) compared with pre-treatment. On the executive function of inhibition, performance significantly improved both at post-test (*p* = 0.016) and 1-month follow-up (*p* = 0.025) compared with pre-treatment. Attention and working memory significantly improved only from pre-treatment to post-treatment (*p* = 0.010). In contrast, processing speed improvement only occurred from pre-treatment to 1-month follow-up (letter sequencing, *p* = 0.031; coding, *p* = 0.017; symbol search, *p* = 0.014) as did verbal learning improvement (*p* = 0.005).

## 4. Discussion

This is the first clinical trial of rTMS to treat MDD in individuals with comorbid HD. Despite HD’s prevalence, treatment options remain limited (Postlethwaite et al., 2019), creating an important unmet clinical need. Behavioral treatment yields only modest improvements in symptoms (18–38%) and minimal research exists on other treatment options (Brakoulias et al., 2015; Rodgers et al., 2021). Psychiatric comorbidities are very common, with over 50% of treatment-seeking individuals with HD also having MDD (Rodgers et al., 2021). This study therefore demonstrates a first step in using rTMS for this difficult-to-treat disorder. There is a clear indication of feasibility for individuals with HD, with preliminary evidence for reductions in MDD and HD symptoms, as well as improvements in cognition.

Using individualized treatment targets optimized from fMRI resting state connectivity between the left dlPFC and SGC, improvements in depression symptoms were observed in 70% of the participants, with 50% achieving clinically meaningful response (50% reduction in depressive symptoms). This rate of response is consistent with typically reported response rates for rTMS for MDD (Brunoni et al., 2017; Liu et al., 2014; Senova et al., 2019). Studies examining rTMS antidepressant effects for MDD have found co-occurring psychiatric disorders to be a risk factor for reduced treatment response (Chou et al., 2021; Trevizol et al., 2020). Most studies demonstrating positive results for MDD with comorbid conditions such as anxiety or PTSD use different treatment targets and protocols (i.e. right sided, low frequency) (Chou et al., 2021; Thompson, 2020). Although our sample was small, the majority of our participants had an additional comorbidity besides MDD + HD, most commonly PTSD or GAD. Our use of individualized *E*-field modeling may have allowed for more effective targeting of MDD-specific neural dysfunction, reducing the confounding effect comorbid diagnoses have on the antidepressant effect of rTMS. Furthermore, we saw maintained or increased antidepressant effects at one-month post treatment. In conjunction with improved cognition, this pattern suggests potential neuroplastic change following rTMS treatment (Appelbaum et al., 2023).

This is the first clinical trial to examine HD symptom changes in response to rTMS treatment. Overall, rTMS to the dlPFC has not been found to be beneficial for anxiety or OCD symptom reduction (Benster et al., 2023) though results are mixed. In this trial there was a 19–29% reduction in HD symptoms with medium effect sizes across timepoints/scales using iTBS to the left dlPFC. These rates of symptom change are similar to improvements in behavioral treatment studies and occurred without any directives about HD behaviors or symptom provocation (Rodgers et al., 2021). These differences in our response rates compared to those for rTMS treatment targeting the dlPFC for OCD or anxiety may be due to the distinct differences in neural response patterns in HD, most often with hypoactivation in the anterior cingulate cortex (ACC) and insula compared to the hyperactivation often seen with individuals with OCD (Mathews et al., 2016; Stevens et al., 2020; Tolin, 2023). Comorbidity rates of HD and MDD are typically at or above 50% across behavioral treatment studies and overlaps in network dysfunction have been suggested but have yet to be thoroughly explored (Levy et al., 2019). However, these findings again support that there may be differences in HD based network dysfunction as compared to OCD as well as suggest a potential overlap between MDD and HD (Kato et al., 2024; Levy et al., 2019; Saxena et al., 2004; Tomiyama et al., 2025).

Prior neuromodulation case studies for HD have focused on right-sided or bilateral treatments. The single case study of rTMS for HD targeted the right dlPFC with low-frequency (≤1 Hz) treatment for thirty sessions and resulted in a 30% decrease on the SI-R (Diefenbach et al., 2015). Two additional case studies have been published using transcranial direct current stimulation (tDCS), another form of noninvasive brain stimulation (Brunelin et al., 2024; Handrack et al., 2018). The first of these tDCS case studies applied direct current bilaterally, using cathodal stimulation to induce neuronal inhibition on the right side, and anodal stimulation to induce neuronal excitation on the left side (Handrack et al., 2018). The second case study targeted only the right side using cathodal inhibitory stimulation (Brunelin et al., 2024). In both tDCS studies, treatment reduced overall HD symptoms by 18.5–24%, and the majority of improvement was driven by reduced acquiring behaviors, with minimal changes to difficulty discarding and no changes in clutter ratings (Brunelin et al., 2024; Handrack et al., 2018). In the current study, we found similar rates of HD symptom reduction, but these reductions were driven by changes in difficulty discarding and clutter, rather than acquiring behaviors. Prior task-based fMRI has found activation in the right dlPFC when making acquiring decisions, while there is bilateral activation of the dlPFC during discarding decisions in individuals with HD (Tolin et al., 2023). Differences in left versus right stimulation targeting and type may differentially target HD symptoms.

There were improvements in key areas of cognitive functioning in individuals with MDD and HD (Fu et al., 2025; Kassel et al., 2024; Mackin et al., 2016; Zakrzewski et al., 2020). Following treatment, there were improvements in scores on visual scanning, inhibition, verbal learning, processing speed, attention, and working memory. These improvements were maintained through 1-month follow-up for all domains except for attention and working memory, which returned to baseline levels. Reviews examining cognitive effects of rTMS for MDD have been mixed in their findings, but most recent meta-analyses have failed to find sustained cognitive improvement (Fu et al., 2025). Treatment studies for HD also have found mixed effects of treatment on cognition (Ayers et al., 2020; Zakrzewski et al., 2020). Further research is needed to determine if these changes are attributable to stimulation, psychiatric symptom reduction, or practice effects.

Despite the novelty of this work, it has several limitations. First, this is a small, open label study and our results demonstrate only the first examination of using rTMS to treat MDD in individuals with HD. These results must be replicated and extended in larger randomized sham-controlled trials before a full understanding of rTMS efficacy in this population can be understood. The use of individualized MDD targets represents the cutting edge of MDD rTMS protocols; however, the variations across individuals in treatment sites make generalization to clinical practice more complex, as neuronavigation is often unavailable in clinical settings. All participants had additional psychiatric comorbidities and while this is typical of HD samples, it is unclear how these additional comorbidities affected treatment response. It is also unclear the potential cause for reductions in HD symptoms, and it is possible these symptom reductions were only a secondary response to depression symptom reductions. Given the small sample size, wide range of age, and multiple comorbidities, we chose to present our data in comprehensive ways, such as mean raw scores and single participant plotting across figures, rather than using more conservative statistical corrections. While this allows for more directly translatable information for future studies, it further emphasizes the need for additional larger samples to confirm our findings. For example, the improvements seen in cognition were not adjusted for multiple comparisons given the sample size as well as the novel and exploratory nature of the study. However, given that many studies do not examine changes in cognition we hope our findings highlight the importance of examining additional factors beyond clinical symptom measures, especially for HD. Finally, the durability of these treatment effects beyond one month are unknown.

This study contributes to a growing literature on effects of brain stimulation beyond diagnoses with FDA indications (Benster et al., 2023). These findings demonstrate the feasibility and initial efficacy of rTMS treatment for patients with MDD + HD and a potential role for rTMS in HD treatment. Due to the lack of evidence-based treatments and clinicians experienced in treating HD, individuals and clinicians often struggle to find and provide adequate care (McGrath et al., 2024; Robertson et al., 2020; Stanley et al., 2017). Using rTMS to treat MDD in individuals with HD may open new avenues of care as well as potential increased motivation or engagement with behavioral treatment for HD due to improved mood (David et al., 2022; Rodgers et al., 2021). Additional research to refine rTMS treatment targets and protocols are needed to improve symptom response and provide wider access to care for individuals affected by this severe and undertreated disorder.

## Supplementary Material

Supplementary Material

## Figures and Tables

**Table 1:** Clinical symptom outcomes.

Domain	Measure	Baseline *Mean (sd)*	Post-Treatment *Mean (sd)*	1-Month Follow-Up *Mean (sd)*	Main Effects for Time
Depression	HAMD	22.4 (3.2)	12.2 (6.2)	11.0 (7.2)	*F*(1.1,10.0) = 15.2, ***p*** = **0.002**, η^2^ = 0.63*
	PHQ9	16.6 (5.0)	11.4 (7.2)	10.4 (8.5)	*F*(1.2,11.2) = 7.3, ***p*** = **0.017**, η^2^ = 0.45*
Hoarding	SIR	63.7 (9.2)	52.2 (18.6)	50.6 (16.6)	*F*(2,18) = 6.7, ***p*** = **0.023**, η^2^ = 0.43*
	SIR: Difficulty Discarding	20.4 (2.3)	16.4 (4.0)	16.2 (3.6)	*F*(2,18) = 16.2, ***p*** < **0.001**, η^2^ = 0.64
	SIR: Clutter	27.6 (5.0)	21.7 (9.1)	20.5 (7.9)	*F*(1.2,10.9) = 7.2, ***p*** = **0.018**, η^2^ = 0.44*
	SIR: Excessive Acquisition	19.4 (3.3)	16.6 (6.8)	16.3 (6.2)	*F*(1.2,10.9) = 2.6, ***p*** = 0.133 η^2^ = 0.23*
	HRS	30.6 (5.3)	12.2 (6.2)	11.0 (7.2)	*F*(2,18) = 7.4, ***p*** = **0.004**, η^2^ = 0.45

* Greenhouse-Geisser Correction; HAMD = Hamilton Depression Scale-17; PHQ9 = Patient Health Questionnaire-9; SIR = Saving Inventory-Revised; HRS = Hoarding Rating Scale-Self Report.

**Table 2:** Significant changes in cognitive performance.

Domain	Measure	Trial	Baseline *Mean (sd)*	Post-Treatment *Mean (sd)*	1-Month Follow Up *Mean (sd)*	Main Effects for Time
Processing Speed	D-KEFS Trail Making Test	Visual Scanning^a^	23.2 (5.3)	20.1 (3.9)	17.9 (3.7)	*F*(1.3,11.5) = 12.5, *p* = 0.003, η^2^ = 0.58*
		Letter Sequencing^a^	34.0 (14.3)	26.1 (5.4)	22.5 (3.7)	*F*(1.2,10.9) = 5.3, *p* = 0.037, η^2^ = 0.37*
	WAIS-IV Processing Speed	Coding^b^	66.6 (15.9)	70.7 (14.6)	75.1 (14.5)	*F*(2,18) = 3.6, *p* = 0.048, η^2^ = 0.29
		Symbol Search^b^	33.2 (9.7)	35.7 (11.2)	38.3 (12.4)	*F*(2,18) = 5.4, *p* = 0.014, η^2^ = 0.38
Executive Functioning	D-KEFS Color Word	Inhibition^a^	58.0 (17.1)	52.2 (15.5)	49.1 (10.5)	*F*(2,18) = 5.4, *p* = 0.016, η^2^ = 0.40
Attention & Working Memory	WAIS-IV Digit Span	Total Score^b^	29.5 (4.7)	33.0 (5.6)	30.7 (4.6)	*F*(2,18) = 6.1, *p* = 0.009, η^2^ = 0.41
Verbal Learning	HVLT-R	Immediate Recall^b^	26.2 (5.0)	28.3 (4.2)	31.4 (1.8)	*F*(2,18) = 6.7, *p* = 0.007, η^2^ = 0.43

All scores listed are means for raw performance.

* Greenhouse-Geisser Correction.

a Improvements marked by reduction in scores.

b Improvements marked by increases in scores; D-KEFS=Delis-Kaplan Executive Functioning System; WAIS-IV = Weschler Adult Intelligence Scale-IV; HVLT-R = Hopkins Verbal Learning Test-Revised.

## Appendix A. Supplementary data

Supplementary data to this article can be found online at https://doi.org/10.1016/j.jad.2026.121814.
