# Supplementary material for: Repetitive transcranial magnetic stimulation (rTMS) for comorbid major depressive disorder and hoarding disorder: An open label pilot study
Source: J Affect Disord. Author manuscript; Available in PMC 2026 May 11. (PMC13159065; doi:10.1016/j.jad.2026.121814)
Supplement: Supplementary Material [file NIHMS2167751-supplement-Supplementary_Material.docx]

**Supplemental Materials**

Table S1: Target x, y, and z coordinates used for treatment by participant

| Participant ID | x | y | z | Coil Orientation |
| --- | --- | --- | --- | --- |
| 01 | -38 | 36 | 28 | 66.9 |
| 02 | -36 | 54 | 18 | 141.1 |
| 03 | -42 | 48 | 18 | 141 |
| 04 | -18 | 24 | 56 | -37.9 |
| 05 | -36 | 12 | 60 | 95.1 |
| 06 | -20 | 30 | 56 | 77.9 |
| 07 | -34 | 30 | 28 | -97.9 |
| 08 | -30 | 48 | 36 | -97.9 |
| 09 | -44 | 32 | 36 | 17.1 |
| 10 | -18 | 34 | 56 | 17.1 |
| 11 | -28 | 44 | 42 | -81.9 |

Table S2: Baseline Neuropsychological Test Performance (Normed Scores)

| **Domain** | **Measure** | **Trial** | **Baseline Normative Score mean scaled score (sd)** |
| --- | --- | --- | --- |
| Estimated I.Q. | WRAT-IV | Word Reading | Standard Score=108 (5.3) |
| Graphomotor Processing Speed | D-KEFS Trail Making Test | Visual Scanning | 10.4 (2.7) |
|  |  | Number Sequencing | 9.9 (4.4) |
|  |  | Letter Sequencing | 11.0 (2.8) |
|  | WAIS-IV Processing Speed | Coding | 10.5 (2.6) |
|  |  | Symbol Search | 11.6 (4.0) |
| Verbal Processing Speed | D-KEFS Color Word | Color Reading | 10.5 (2.7) |
|  |  | Word Reading | 10.9 (3.0) |
| Executive Functioning: Set Shifting & Inhibition | D-KEFS Trail Making Test | Switching | 11.3 (1.6) |
|  | D-KEFS Color Word | Inhibition | 10.0 (3.4) |
|  |  | Inhibition/Switching | 10.4 (3.9) |
| Attention & Working Memory | WAIS-IV Digit Span | Total Score | 11.3 (2.1) |
| Attention |  | Forward Span | 12.8 (2.4) |
| Working Memory |  | Backward Span | 9.8 (2.1) |
|  |  | Sequencing | 9.8 (2.2) |
| Verbal Learning | HVLT-R | Immediate Memory | 9.0 (3.6) |
| Verbal Memory |  | Delayed Memory | 9.5 (3.2) |
|  |  | Retention Percentage | 10.1 (2.3) |

Standard Scores have a mean of 100 and standard deviation of 15. Scaled score range from 1-19 with a mean score of 10 and a standard deviation of 3. D-KEFS= Delis Kaplan Executive Functioning System; WAIS-IV= Weschler Adult Intelligence Scale; HVLT-R=Hopkins Verbal Learning Test-Revised
